# Exposure to Depression Memes on Social Media Increases Depressive Mood and It Is Moderated by Self-Regulation: Evidence From Self-Report and Resting EEG Assessments

**DOI:** 10.3389/fpsyg.2022.880065

**Published:** 2022-06-29

**Authors:** Atakan M. Akil, Adrienn Ujhelyi, H. N. Alexander Logemann

**Affiliations:** ^1^Doctoral School of Psychology, ELTE Eötvös Loránd University, Budapest, Hungary; ^2^Institute of Psychology, ELTE, Eötvös Loránd University, Budapest, Hungary

**Keywords:** depression memes, EEG, emotion regulation, frontal alpha asymmetry, internet, social media, self-regulation

## Abstract

This study aimed to investigate the effects of depression memes, spread mainly on social media, on depressive mood, and the moderating role of self-regulation based on self-report and electrophysiological (resting EEG frontal alpha asymmetry) assessments. We conducted a semi-online crossover study; first, we collected brain activity data from healthy young adults (*n* = 32) who were subsequently provided a link to the online experiment. Each participant participated in both the neutral and meme conditions. We also evaluated their level of depressive mood immediately before and after exposure to the stimuli. We further conducted a series of linear mixed effects model analyses and found that depression memes contributed to an increase in depressive symptoms. Specifically, lack of emotional clarity, difficulties in goal-directed behaviors in emotional distress, and impulse control difficulties were linked to greater depressive mood in the case of exposure to depression memes compared with neutral images. However, time interactions were insignificant. These results mainly indicate the centrality of behavioral problems during times of emotional distress caused by depression memes. Lastly, although frontal alpha asymmetry did not predict a change in depressive mood or significantly differ across conditions, lower inhibitory control may result in increased processing of depression memes as negative stimuli. This result is consistent with our self-report results (e.g., impulsivity) as well as other related studies in the literature. However, further research is needed to verify these frontal alpha asymmetry results.

## Introduction

Depression is among one of the most prevalent mood disorders in the world. The number of individuals who are suffering from depression is more than 264 million including all ages worldwide and it has been declared as “a major contributor to the overall global burden of disease” (World Health Organization, 2020). Therefore, depression has a great deal of influence on people’s lives, affecting them in many ways, such as quality of life (Wang et al., 2017). Some of the symptoms of depression include sadness, hopelessness, sleeping problems, worthlessness, and suicidal ideation (American Psychiatric Association, 2013).

Recent technological innovations in communication have made depression more apparent in daily life. For instance, participative (or people-centric) Internet technologies, such as social media platforms, facilitate collaborative content creation and moderation for individuals without time or space restrictions (Aghaei et al., 2012; Choudhury, 2014). Therefore, such technologies also provide an environment for people suffering from depression or depressive mood to share their emotions. Furthermore, not just the visibility of depression-related content, but also the form of expressing depression has also changed following these advancements. Depression memes are a new form of communication used on the Internet (Image 1) [Please check our online repository for Image 1: https://osf.io/b5ep9].

The term meme, short for mimeme, an Ancient Greek word meaning that is “imitated” or “imitated thing” was coined by the evolutionary biologist Richard Dawkins and introduced in his internationally popular book “The Selfish Gene” (Dawkins, 1976). He defined memes as cultural units that spread information in a gene-like fashion among people. Some examples of memes are ideas, catchphrases, tunes, fashion, and building arches. Just as genes are passed on from body to body as replicators, memes are also replicators passed on from brain to brain. For instance, when a scientist reads or hears about an idea, they convey it to their colleagues and students through articles or lectures. When an idea becomes popular, it can be said to propagate itself and passed on from brain to brain in the form of a meme (Dawkins, 1976).

Evidently, the term “meme” was conceived long before the present Internet era; however, it has been revived by Internet users. Although these two types of memes are closely related, Internet memes are defined slightly differently from the traditional understanding of memes: digital items that share common characteristics in form and content and are disseminated by many Internet users (Shifman, 2014, p. 41). Similarly, Jenkins et al. (2013) characterized Internet memes as “spreadable media.” Therefore, the concept of the memes is based on the idea that information, whether in the form of a gene, text, or image is interested in one thing which is to be spread wide and far (Miltner, 2017).

Miltner (2014) and Milner (2016) argued that Internet memes (hereinafter referred to as memes) have become popular as they evoke emotional resonance; people like and share memes owing to their emotional engagement with them. Specifically, depression memes are commonly shared in an attempt to express feelings that are, generally negative; therefore, when individuals interact with these memes, they are influenced by the messages that reach them, which need special attention and consideration. Currently, a Google search for “depression memes” yields more than 350,000,000 results. There are many social media accounts of aggregated depression memes with thousands of followers (e.g., depression_memes subreddit on Reddit.com has currently more than 215,000 followers). Hence, these memes are easily accessible for everyone even though some social media platforms send warning messages about them. For instance, when “depression” is searched on Instagram, the system sends a message saying “*Posts with words or tags you are searching for often encourage behavior that can cause harm and even lead to death.*” On the other hand, it is easy to pass this message by just clicking on “see results” button.

The increasing use of depression memes has resulted in an effort to investigate them. However, we realized that this new phenomenon is still understudied in scientific literature. In one of the few related studies, Jadayel et al. (2018) found in their case study that depression memes may encourage people to physical harm and suicidal thoughts but Akram et al. (2020) discovered in their exploratory study that humorous depression memes may be beneficial for individuals in case adaptive emotion regulation strategies are used. While the scientific community has relatively ignored the topic, it is widely discussed in popular media, for example, in recently published articles entitled “Can Memes Benefit Mental Wellness?” in Psychology Today (Ali, 2021) and “Sharing Mental Health Memes Is Making Things Worse, Not Better” in Refinery 29 (Morgan, 2021).

Evidently, depression memes can vary in their emotional tones (Image 1). While some depression memes are humorous, which account for one of the most frequent types, and can cause laughter, others are more negative with sad quotes and black and white, darker depictions of suffering people (Image 2) [Please check our online repository for Image 2: https://osf.io/b5ep9]. According to the literature, the effects of negative depression memes on depression-related symptomatology and the moderating role self-regulation are still unknown. Therefore, we aimed to examine negative depression meme (hereinafter referred to as depression meme) in emotional processes and compare the role of self-regulation evaluated subjectively and objectively. Specifically, this study investigated the moderating role of self-report and brain activity reflections of self-regulation on the effects of exposure to depression memes on depressive symptoms.

Besides depression and memes, self-regulation is another important concept in this study that has to be explained in detail before proceeding. Self-regulation is inextricably associated with human life and it helps individuals to regulate their emotions, thoughts, and behaviors and not get overwhelmed by negative and stressful life events. Therefore, it is crucial for everyday functioning (Heatherton and Wagner, 2011). There are two main manifestations of the regulatory system; approach and avoidance motivations, which are considered dependent on environmental properties (Amodio et al., 2008; Robinson et al., 2016). Specifically, cues that signal reward provoke approach motivation while cues that signal threat invoke avoidance/withdrawal motivation, which accounts for inhibitory control. Hence, this mechanism allows people to cope with rewarding and threatening stimuli that arise in their environment.

In the 1970s, these two motivational systems were found to be associated with the asymmetry of frontal brain activity. Ongoing studies have elucidated the role of frontal hemispheric asymmetry based on the alpha (8–13 Hz) signature of electroencephalography (EEG) in manifesting individual differences in motivational orientations and affect measures (Davidson et al., 1979; Palmiero and Piccardi, 2017; Allen et al., 2018). Frontal alpha asymmetry is a relative measure of the right and left alpha activity in the frontal regions of the brain. Specifically, lower frontal alpha asymmetry scores (calculated right minus left alpha activity), indicate relatively less alpha activity in the right relative to the left region, resulting in an enhanced right frontal cortex (avoidance tendency toward negative stimuli) relative to the left (approach tendency toward positive stimuli; Coan and Allen, 2004; Harmon-Jones et al., 2010). Therefore, successful self-regulation is dependent on top-down control over the frontal regions. Impaired frontal alpha activities would tip the balance of the regions, which has been shown to be an essential reason for poor self-regulation (Heatherton and Wagner, 2011). However, previous studies have also reported contradictory results regarding the relationship between frontal alpha asymmetry, depression, and self-regulation. For example, some studies found that lower frontal alpha asymmetry (higher avoidance motivation) is associated with more negative evaluations of all stimuli and diminished emotional modulation (Adolph et al., 2017). On the other hand, other studies found that compared with healthy individuals, adolescents with major depression exhibited less left-sided alpha activity (higher approach motivation; Grünewald et al., 2018) and no difference in frontal alpha asymmetry (Feldmann et al., 2018). Moreover, in their meta-analysis, van der Vinne et al. (2017) found that frontal alpha asymmetry is questionable and is not a reliable biomarker of depression. Therefore, this study considers frontal alpha asymmetry as an index of self-regulatory tendencies (i.e., approach and avoidance/withdrawal) at the exploratory level, rather than that of depression.

Altogether, we expected that, relative to neutral images, depressive mood would generally increase after exposure to depression memes in the event of poor emotion regulation during the emotional process. We also explored the moderating role of dispositional frontal alpha asymmetry in the relationship between exposure to depression memes and depressive symptoms.

## Materials and Methods

### Design

We implemented a semi-online crossover design. We invited the participants to our laboratory for the collection of brain activity data in the context of a large comprehensive project. Subsequently, they were provided a link to the online experiment through which they could participate in the research remotely. Each participant was exposed to both neutral images and depression memes. To avoid carry-over effects, we counterbalanced the order of the stimulus sets; therefore, half of the participants started with the neutral condition. Furthermore, we gave a break of at least one day as a wash-out period between the two conditions. Stimuli in the given condition were presented randomly. Depressive symptoms were assessed immediately before and after each intervention. Our main variables were eyes closed/eyes open frontal alpha asymmetry, collected in the laboratory as an objective measurement of self-regulation; emotion regulation skills based on a questionnaire as a subjective measurement; and repeated measures of depressive moods collected before and after the stimuli presentation.

### Participants

Participants were recruited *via* social media ads. Our inclusion criteria included being at least 18 years old and exclusion criteria included having psychological/psychiatric disorder(s), frequent headaches/migraines, epilepsy, past significant head trauma, recent head trauma, and current drug use. The participants abstained from smoking and drinking alcohol or coffee prior to the EEG assessments. As mentioned before, participants visited our laboratory at ELTE Eötvös Loránd University for the brain activity data, and subsequently, they participated in the online part of the study remotely in the following days. Our sample consisted of 32 people (20 female (%62.5) and 12 male (%37.5); *M*_age_ = 29.4 years, *SD* = 9.5, Min_age_ = 21, Max_age_ = 59). All participants were informed about the procedure before the experiment. It was emphasized that the experiment contains depression-related stimuli and asked them not to continue if they have elevated sensitivity toward such stimuli. All participants provided their informed consent prior to any procedures. At the end of the experiment, necessary websites and hotlines were provided for their safety. Furthermore, they could always contact the responsible investigator and were provided with the possibility of assistance if they experienced any discomfort subsequent to the experimental procedures. The study was approved by the Ethics Committee at ELTE Eötvös Loránd University (reference numbers: 2020/403 and 2021/314) and it was conducted in accordance with the Declaration of Helsinki and its later amendments.

### EEG Recordings and Analyses

Electrophysiological activity was recorded by using the Mind Media NeXus-32^1^ amplifier in combination with a 21-channel cap with Ag/AgCl electrodes. A 21-channel cap was used because the brain activity was collected in the context of a large comprehensive project, and because of the availability of the NeXus-32 system in our laboratory, which made it a feasible approach. In addition, this multichannel approach is relatively common (Peeters et al., 2014; Smith et al., 2017) and it allows for redundancy. The data were recorded at a sampling rate of 512 using the common average. The horizontal electrooculography (HEOG) was recorded bipolarly from the outer canthi of both eyes, and the vertical EOG (VEOG) was recorded above and below the left eye. Recorded data were re-referenced to linked mastoids, and a low cutoff filter of 0.5 Hz and a high cutoff filter of 40 Hz was employed. Following that, the first and last 10 s of the EEG data was excluded because these generally contain artifacts. Subsequently, data were further segmented in 2-s epochs. These epochs were corrected for ocular artifacts, based on VEOG and HEOG electrodes, by using independent component analysis (ICA). Epochs containing remaining artifacts, based on min–max 75 microvolt amp. Criterion, were discarded. Remaining epochs were whole-segment baseline corrected, and Power Spectral Density (PSD) was calculated by using FFT with a 10% Hanning window. Afterward, the epochs were averaged, and power, mean activity, in the alpha frequency band (8–13 Hz) was calculated and values exported for the relevant electrodes. By using the Statistical Package for Social Sciences (SPSS; IBM Corp, 2019), alpha power was corrected for skew *via* natural log transform (Smith et al., 2017). Lastly, the frontal asymmetry was calculated *via* subtraction of log-transformed alpha at lateral left electrode sites from right electrode sites.

### Stimuli

We used a depression memes set validated in another study (Akil et al., 2022; Unpublished manuscript)^2^. This set consisted of depression memes from Tumblr.com, a microblogging and social networking website, selected with the help of the Diagnostic and Statistical Manual of Mental Disorders (DSM-5) for Major depressive disorder (MDD; American Psychiatric Association, 2013). We used the same memes (*n* = 10) that has also been used in this study (Image 2). We did not fix the duration of the stimuli because the text, sad quotes, in them may require different cognitive and linguistic skills to understand. We did not change the size of them [approximately on average: 408 (w) × 320 (h)] and grammatical errors in those stimuli because they are all unique. Indeed, this makes our experiment ecologically valid because our stimuli were not designed artificially for this experiment and they reflect real-life circumstances. For the neutral condition, we used neutral images, not memes, (*n* = 10) from the Geneva Affective Picture Database (GAPED; Dan-Glauser and Scherer, 2011). These neutral images mainly depict inanimate objects.

### Questionnaires

Self-report assessments were implemented in PsyToolkit,^3^ a free-to-use toolkit for programming and running cognitive-psychological experiments and surveys (Stoet, 2010, 2017).

#### Common European Framework of Reference for Languages—Self-Assessment Grid

This question was used for the exclusion of participants who had lower than B1 level English during the analyses. We asked them the following question: “Please indicate your English language proficiency” and six response options indicated the levels of language proficiency; *A1 Basic User*, *A2 Basic User*, *B1 Independent User*, *B2 Independent User*, *C1 Proficient User*, and finally *C2 Proficient User*. B1 Independent User represents that “I can understand texts that consist mainly of high frequency everyday or job-related language. I can understand the description of events, feelings, and wishes in personal letters.” After the analysis, none of the participants excluded because of this criterion.

#### Difficulties in Emotion Regulation Scale-16

The Difficulties in Emotion Regulation Scale (DERS) was developed by Gratz and Roamer (2004) to measure emotion regulation deficits. Bjureberg et al. (2016) shortened the questionnaire and created the DERS-16. There are five subscales in the questionnaire; *clarity* (measures lack of emotional clarity); *goals* subscale (measures difficulties in goal-directed behaviors in the context of emotional distress); *impulse* subscale (measures impulse control difficulties); non-acceptance (measures non-acceptance of emotional responses); and lastly, *strategies* (measures limited access to adaptive emotion regulation strategies). Each subscale consisted of three items scored on a 5-point Likert scale ranging from “almost never” to “almost always.” The relevant outcomes for each subscale were sum scores of the items and higher scores represented more difficulties in emotion regulation whereas lower scores less difficulty. We found relatively high internal consistency (Cronbach’s alpha) for each subscale: clarity was 0.90; goals 0.82; impulse 0.94; non-acceptance 0.87; and strategies 0.84.

#### Profile of Mood States-Short Form

The Profile of Mood States (POMS) was created by McNair et al. (1971) to measure mood state. It was shortened by Shacham (1983) and validated with a group of cancer patients. Based on this shortened version, Curran et al. (1995) validated the form for different groups including healthy adults. The POMS-Short Form (SF) is an adjective checklist consisting of 37 items rated on a 5-point Likert-type scale that ranges from “not at all” to “extremely.” We considered only depression subscale because our stimuli set did not match with the other subscales conceptually (e.g., anger and vigor). This subscale consisted of eight items (e.g., unhappy, hopeless, and worthless) measuring various symptoms of depression and giving overall depressive mood score. In the present research, internal consistencies (Cronbach’s alphas) were 0.89 for pre-intervention and 0.95 for post-intervention and considered as high.

### Procedure

**Figure 1:**
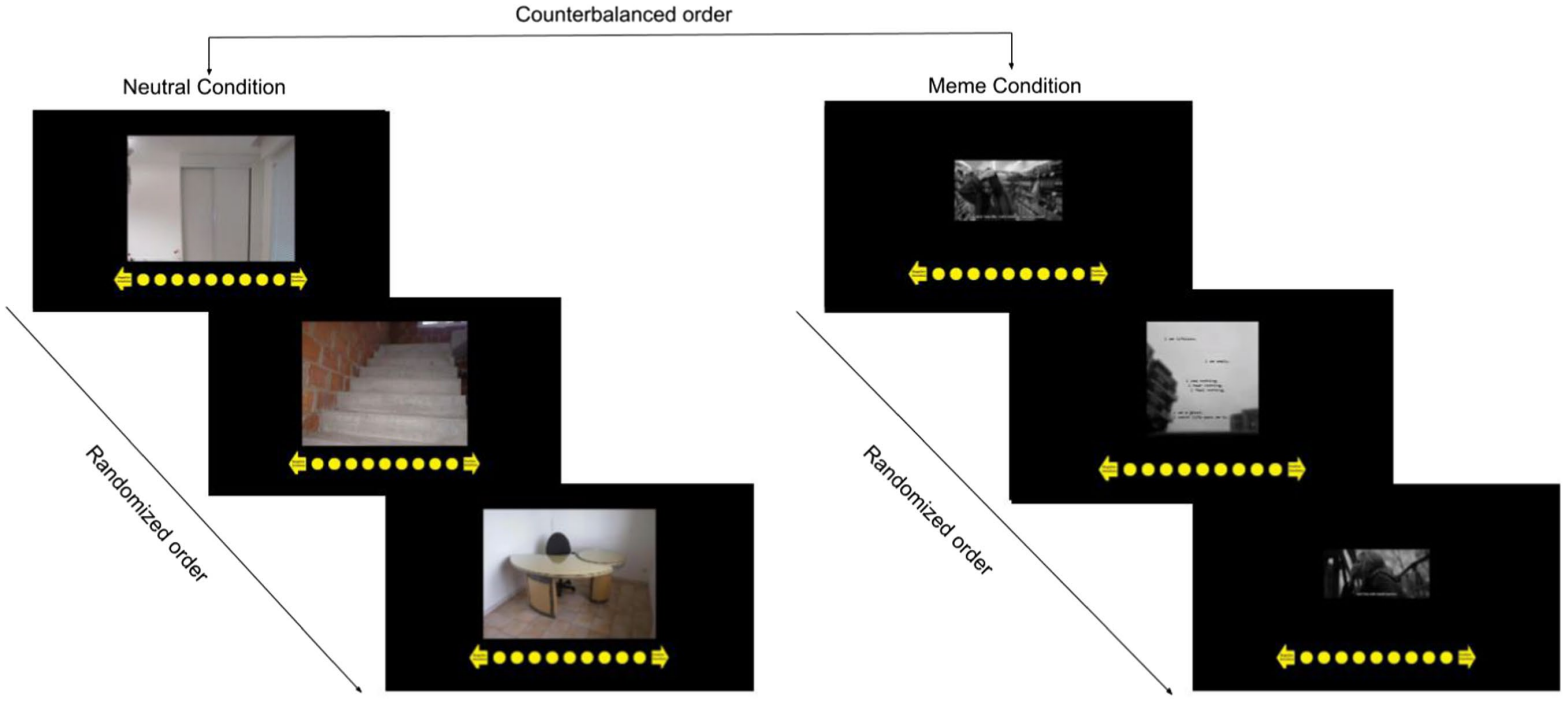
An example of the procedure with counterbalanced condition order and randomized stimuli order.

First, when participants arrived at our laboratory, after reading the information letter, checking the exclusion criteria, and signing the informed consent form, they were seated in a comfortable chair in a dimly lit testing room for the placement of the EEG cap and ocular electrodes. Following electrode placement, we collected the resting-state EEG data. To reduce muscle artifacts in the EEG signal, the participants were instructed to avoid movement and excessive eye blinking. The resting-state EEG was recorded for 10 min with 5 min eyes open and 5 min eyes closed blocks. These electrophysiological data from a comprehensive laboratory project were used for the calculation of frontal alpha asymmetry in the current study, and because of feasibility reasons, the online experiment was performed in the following days of the EEG assessments. In the second part of the study, the participants were provided with a link for remote online participation. The participants were provided instructions about this part, during which they gave informed consent again. The online experiment encompassed two sessions separated by at least one day. The sessions differed with respect to the valence of the stimuli, either neutral or depressed (Figure 1). The order of the stimuli was counterbalanced across the participants. For each session, the procedure was as follows. First, participants filled out the demographic questions, DERS-16, and POMS-SF. Subsequently, the stimuli, either neutral images or depression memes, were presented randomly in the middle of the screen, after which, the participants were asked to indicate whether the stimulus was negative or positive on a 9-point Likert scale, in order to retain their attention on the stimulus and avoid potential distractions in their environment. Finally, the participants filled out the POMS-SF again to evaluate post-intervention depressive mood, which completed the session. The research was then concluded in the second session. The complete procedure lasted approximately 20 min.

### Statistical Analyses

We used SPSS (IBM Corp, 2019) for the following preliminary steps of the analyses. Both the resting EEG and self-report data sets were merged. The sum scores of the questionnaires were calculated by adding up the items for each subscale. In the final data set, “C1” represents the neutral condition while “C2” represents the meme condition (e.g., the variable “C1post” means neutral condition post-depressive mood). The main analyses were conducted *via* R (R Development Core Team, 2017). Because we had a crossover design with repeated measures, we chose linear mixed effects model analysis due to some advantages over traditional repeated measures analysis of variance (ANOVA). First of all, linear mixed effects models have been developed to take into consideration of nested (multiple observations within a subject in a given condition) and crossed (subjects in multiple conditions) structure of data (Boisgontier and Cheval, 2016) and thereby, gives results with an acceptable rate of Type 1 error (i.e., acceptable reliability; Baayen et al., 2008). Furthermore, linear mixed effects models are resistant to assumption violations (Schielzeth et al., 2020) and they allow incomplete and unbalanced data to be used. Therefore, we did not exclude any outliers because they are not necessarily erroneous values. Our models were “Depression ~ Self-regulation (Covariate) × Time × Condition + (1|ID).” All self-regulation predictors (i.e., clarity, goals impulse, non-acceptance, strategies, and frontal alpha asymmetry) were numeric variables. As can be seen from the example model, within and between individual variability were the random effects. Lastly, we conducted post hoc analyses for the significant results. All data, codes, and the analysis results can be found *via* our online repository.

### Results

Descriptive statistics of the main variables in the experiment is displayed in Table 1.

**Table 1:** Descriptive statistics of the main variables in the experiment.

Variables		*M*	Min	Max	*SD*
Neutral Condition					
	Pre-depressive mood	13.68	8	40	7.17
	Post-depressive mood	13.46	8	40	7.17
Meme Condition					
	Pre-depressive mood	13.46	8	30	5.36
	Post-depressive mood	15.18	8	38	7.83
Clarity		4.31	10	10	1.78
Goals		8.37	3	15	3.08
Impulse		6.15	3	15	3.35
Non-acceptance		6.59	3	15	3.46
Strategies		10.53	5	22	4.66
FAA EO		−0.04	−1.49	0.39	0.34
FAA EC		0.002	−0.21	0.37	0.10

*Abbreviations: FAA: Frontal alpha asymmetry; EO: Eyes open; EC: Eyes closed.*

Table 2 shows that there is no carry-over effect between the sessions. Specifically, there is no significant difference between the neutral condition pre-depressive mood (*M* = 13.68, *SD* = 7.17) and meme condition pre-depressive mood (*M* = 13.46; *SD* = 7.17); *t*(31) = 0.19, *p* = 0.850. This indicates that at least a day wash-out break between sessions resulted in the pre-experiment depressive mood level. Moreover, the table indicates a significant difference between pre-depressive mood (*M* = 13.46; *SD* = 5.36) and post-depressive mood (*M* = 15.18; *SD* = 7.83) in the meme condition; *t*(31) = −2.08, *p* = 0.045 whereas there was no significant difference between pre-depressive mood (*M* = 13.68; *SD* = 7.17) and post-depressive mood (*M* = 13.46; *SD* = 7.17) in the neutral condition; *t*(31) = 0.57, *p* = 0.567, which shows a successful manipulation.

**Table 2:** Paired samples *t*-test results for carry-over effects and manipulation check.

Variables				*M*	*SD*	Lower CI	Upper CI	*t*	*df*	*P*
Neutral Condition										
Pre–Post-depressive mood				0.21	2.13	−0.55	0.98	0.57	31	0.567
Meme Condition										
Pre–Post-depressive mood				−1.71	4.66	−3.40	−0.03	−2.08	31	0.045*
Inter-condition										
(Neutral) Pre(Meme)–Pre-depressive mood				0.21	6.47	−2.11	2.55	0.19	31	0.850
(Neutral) Post(Meme)–Post-depressive mood				−1.5	9.23	−4.83	1.83	−0.91	31	0.365

*Significance level used: 0.05.

*Confidence level used: 0.95.*

As mentioned earlier, we conducted a series of mixed effect model analyses for the moderating role of emotion regulation in the relationship between the effects of depression memes on depressive mood. The results are presented in Table 3.

**Table 3:** Results of linear mixed effects models for self-report emotion dysregulation.

Variables/Models				Num *df*	Den *Df*	*F*	*P*
**Clarity**				1	30	28.56	<0.001*
Time				1	90	0.50	0.48
Condition				1	90	5.19	0.025*
Clarity × Time				1	90	1.32	0.253
Clarity × Condition				1	90	8.09	0.005*
Clarity × Time × Condition				1	90	0.45	0.500
**Goals**				1	30	6.15	0.018*
Time				1	90	0.24	0.618
Condition				1	90	11.86	<0.001*
Goals × Time				1	90	0.78	0.377
Goals × Condition				1	90	16.15	<0.001*
Goals × Time condition				1	90	1.41	0.237
**Impulse**				1	30	3.54	0.069
Time				1	90	0.01	0.894
Condition				1	90	3.24	0.074
Impulse × Time				1	90	0.42	0.517
Impulse × Condition				1	90	6.47	0.012*
Impulse × Time condition				1	90	0.86	0.354
**Non-acceptance**				1	30	13.85	<0.001*
Time				1	90	0	0.956
Condition				1	90	4.56	0.035*
Non-acceptance × Time				1	90	0.16	0.682
Non-acceptance × Condition				1	90	3.73	0.056
Non-acceptance × Time × Condition				1	90	0.06	0.803
**Strategies**				1	30	9.47	0.004*
Time				1	90	0.20	0.652
Condition				1	90	0.20	0.652
Strategies × Time				1	90	0.01	0.909
Strategies × Condition				1	90	3.73	0.056
Strategies × Time × Condition				1	90	0.04	0.830

*Dependent variable: Depressive mood.*

*Significance level used: 0.05.

**Figure 2:**
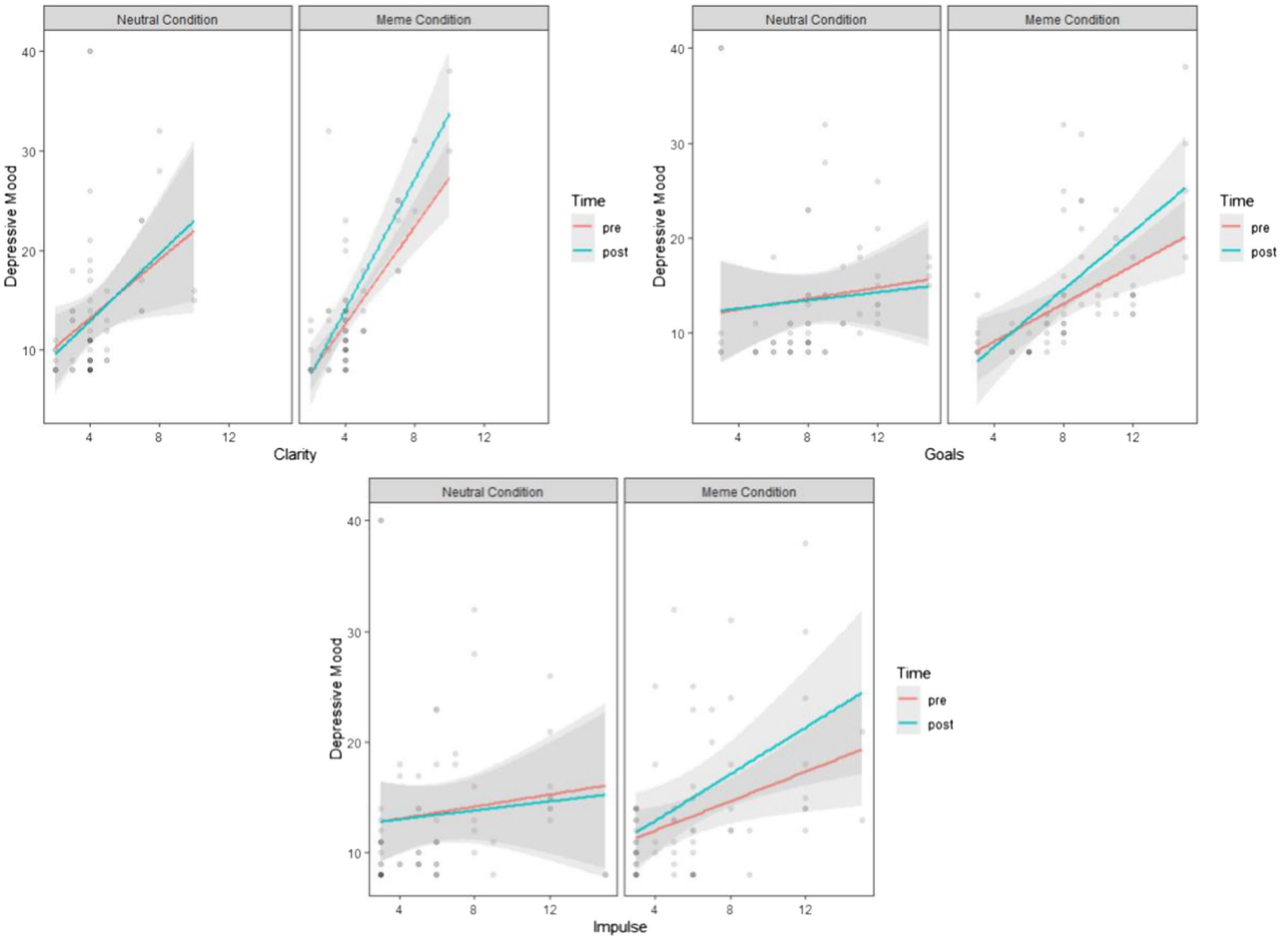
The effects of depression memes on depressive mood, compared with neutral images, when the covariate is lack of emotional clarity, difficulties in goal-directed behaviors during emotional distress, and impulse control difficulties.

As can be seen from Table 3, depressive mood and condition interaction was significant for most of the models; clarity, goals, and non-acceptance, but the time interactions were insignificant. Specifically, depressive mood differs significantly based on the conditions and their interactions with lack of emotional clarity, *F*(1,90) = 8.09, *p* = 0.005; difficulties in goal-directed behaviors during emotional distress, *F*(1,90) = 16.15, *p* = 0.001; and difficulties in impulse control, *F*(1,90) = 6.47, *p* = 0.012. Therefore, these results reveal that depressive mood is moderated by the maladaptive emotion regulation strategies in the case of exposure to depression memes, compared with neutral images, as depicted in Figure 2.

**Figure 3:**
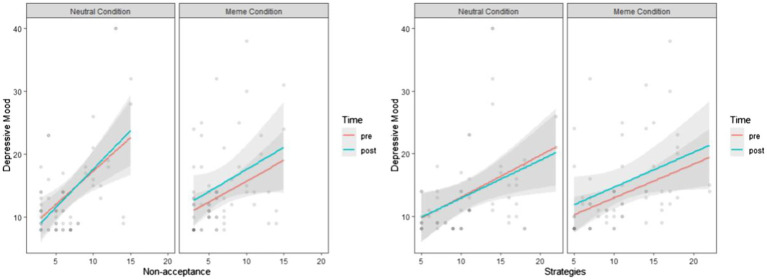
The effects of depression memes on depressive mood, compared with neutral images, when the covariate is non-acceptance of emotional responses and limited access to adaptive emotion regulation strategies.

Although the results were insignificant, the figures showed similar patterns for non-acceptance of emotional responses and limited access to adaptive emotion regulation strategies, as illustrated in Figure 3.

#### Post Hoc Analyses

We performed a series of pairwise comparisons (Tukey adjusted) to verify the significance of the slopes of clarity, goals, and impulse visualized in Figure 2. Table 4 shows the results.

**Table 4:** Results of pairwise comparisons of clarity, goals, and impulse slopes by condition.

Variables			Trend	*SE*	Lower CI	Upper CI	Estimate	*T*	*p*
**Clarity**									
Neutral condition			1.56	0.47	0.61	2.51			
Meme condition			2.85	0.47	1.90	3.80			
Contrast							−1.29	−2.84	0.005*
**Goals**									
Neutral condition			0.24	0.32	−0.41	0.91			
Meme condition			1.26	0.32	0.59	1.92			
Contrast							−1.01	−4.02	<0.001*
**Impulse**									
Neutral condition			0.23	0.31	−0.40	0.87			
Meme condition			0.85	031	0.22	1.49			
Contrast							−0.62	−2.54	0.012*

*Significance level used: 0.05.

*Confidence level used: 0.95.*

**Figure 4:**
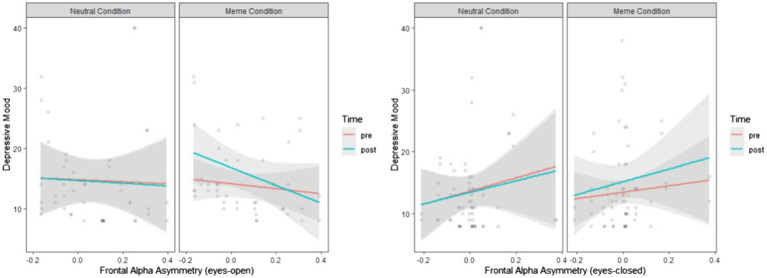
The effects of depression memes on depressive mood compared with neutral images when the covariate is eyes open and eyes closed frontal alpha asymmetry as a neural marker of self-regulation.

Table 5 shows the linear mixed effects models results for the effects the effects of depression memes, compared with neutral images, on depressive mood and the moderating role of frontal alpha asymmetry. Similar to non-acceptance and strategies results, the effects were not significant despite showing a similar pattern, as illustrated in Figure 4, compared to the other self-reported covariates.

**Table 5:** Results of linear mixed effects models for frontal alpha asymmetry.

Fixed effects			Num *df*	*Den df*	*F*	*p*
FAA EO			1	26	0.27	0.607
Time			1	78	0.46	0.496
Condition			1	78	0	0.98
FAA EO × Time			1	78	0.07	0.787
FAA EO × Condition			1	78	0.47	0.490
FAA EO × Time × Condition			1	78	0.13	0.716
FAA EC			1	30	0.86	0.359
Time			1	90	0.78	0.376
Condition			1	90	0.81	0.369
FAA EC × Time			1	90	0.05	0.811
FAA EC × Condition			1	90	0.07	0.782
FAA EC × Time condition			1	90	0.16	0.685

*Dependent variable: Depressive mood.Significance level used: 0.05.4 participants were excluded from the FAA EO model due to missing values.*

## Discussion

This study examined the effects of depression memes on social media on depression-related symptoms (e.g., sadness, unhappiness, hopelessness, and worthlessness based on a depression scale reflecting an overall depressive mood score), and the moderating role of self-regulatory skills. In the context of Internet usage, self-regulation is considered crucial for upregulating positive and downregulating negative emotions (LaRose et al., 2001). Accordingly, we found that, compared with neutral images, higher disposition toward maladaptive emotion regulation strategies, in other words emotion dysregulation, especially lack of emotional clarity, difficulties in goal-directed behaviors during emotional distress, and impulse control difficulties, result in greater depressive mood in the case of exposure to depression memes, and possibly reduce the quality of life. These significant results may be attributable to the centrality of impulsivity in emotion regulation problems. Previous studies have shown that impulse disorder is the main behavioral dysfunction in various emotion-related problematic behaviors and mental diseases (Tice et al., 2001; Zhang et al., 2020). Therefore, compared with neutral images, depression memes are more harmful for individuals suffering from difficulties in goal-directed behaviors during emotional distress and from impulse control difficulties. These results are consistent with those of other studies that investigated the effects of negative stimuli, rather than depression memes, and the role of emotion regulation (Erk et al., 2010; Joormann and Quinn, 2014; Speed et al., 2020). Additionally, depression memes also resulted in increased depressive mood in the case of a lack of emotional clarity. According to the literature, deficits in emotional clarity also result in maladaptive responses and increased depressive symptoms (Flynn and Rudolph, 2010; Blöte and Westenberg, 2019).

In contrast, better emotion regulation skills, seem to allow people to make light of the negative experiences of depression memes. In other words, changes in depressive mood after exposure to depression memes compared with neutral images may potentially be moderated by deficits in the ability to employ adaptive emotion regulation strategies, such as positive reappraisal. However, a more comprehensive outcome of the effects of emotion regulation in the case of exposure to depression memes could be possible with the direct application of adaptive emotion regulation strategies to the models.

We also considered frontal alpha asymmetry as a neural marker of self-regulatory motivations and investigated its moderating role in the relationship between exposure to depression memes and depressive symptoms. Despite the affective nature of depression memes, the results were statistically insignificant. The statistical power to identify such a moderating effect is attributable to the restricted range of frontal alpha asymmetric oscillatory patterns in our sample. In this vein, it is important to note that our sample comprised healthy and educated young adults. Therefore, their frontal alpha asymmetry scores may have been too homogenous to yield significant results. However, the insignificant results do not necessarily mean that the variables are not related.

The results regarding the predictive role of frontal alpha asymmetry in changes in depressive mood after exposure to depression memes can indicate several factors. Particularly, eyes closed frontal alpha asymmetry showed a similar pattern to our subjective evaluation of maladaptive emotion regulation strategies, such as difficulties in goal-directed behaviors in emotional distress and impulse control difficulties in case it is considered a lower inhibitory control, with a higher frontal alpha asymmetry score. Therefore, our results are consistent with those of studies that indicate that inhibitory control deficits result in increased processing of negative stimuli (Gotlib and Joormann, 2010; Disner et al., 2011; García-Martín et al., 2021); this is vital for emotion-related problems because it allows individuals to limit unwanted behaviors, thoughts, and emotions and provides flexibility for adapting to diverse environmental contingencies and specific goals (Anderson and Weaver, 2009). However, a higher frontal alpha asymmetry score also means less alpha activity in the left frontal cortex, that is, higher approach motivation to positive stimuli. In this case, our results were not sufficiently indicative. Specifically, eyes open frontal alpha asymmetry showed that higher avoidance/withdrawal tendency or inhibitory control, as indexed by the lower frontal alpha asymmetry scores, results in lower depressive mood after exposure to depression memes compared with neutral images, consistent with previous studies (Coan and Allen, 2004; Harmon-Jones et al., 2010).

However, the results indicate that complex relationships between frontal alpha asymmetry, self-regulation, and depression may be a potential reason for the inconsistent findings of frontal alpha asymmetry studies. As mentioned earlier, related studies have found contradictory results regarding frontal alpha asymmetry and its relationship with depression (Kołodziej et al., 2021) and self-regulation (Coan and Allen, 2003; Jesulola et al., 2015). An ideal way to address these issues would be to directly manipulate the asymmetry of frontal brain activity and assess event-related potentials (ERPs) with respect to behaviors, cognition, and/or emotions. In conclusion, our results regarding frontal alpha asymmetry can also be attributable to a chance factor as they are insignificant.

This study has several limitations. First, the sample comprised young healthy adults; as such, the present findings may only be generalizable to populations with the same features. We suggest that further studies examine individuals that have been clinically diagnosed with depression or use a comprehensive assessment for depressive symptoms from the perspective of diagnostic criteria. Therefore, this study cannot be extrapolated to high-risk individuals. Moreover, we found that depression memes are harmful even to healthy and educated young adults. Therefore, it is also important to examine other sample groups, such as adolescents, in this context. We performed investigations immediately before and after participants’ exposure to depression memes. It is important to further consider the depression memes’ long-term effects on depression. For instance, constant exposure to these memes is highly probable owing to the algorithms that are used by social media platforms. Moreover, the effects of depression memes can potentially be more negative with maladaptive strategies in the long term. In conclusion, besides maladaptive self-regulation strategies, it could also be important to add adaptive strategies; in this way, the exact difference they make in depressive mood could be noted. Therefore, there is a need for a replication study eliminates these limitations.

Additionally, besides these limitations, future studies can consider employing new approaches. For example, we assessed depressive symptoms using a self-report questionnaire. There are, however, new automated computerized depression detection methods based on EEG signals (Akbari et al., 2021a,b) that can be used for a more objective evaluation of depressive symptoms. Moreover, depression can be comorbid with various neuropsychological/neuropsychiatric diseases; therefore, future studies can consider employing network approaches and more comprehensive frameworks in their models (Sadiq et al., 2021; Yu et al., 2022).

## Data Availability Statement

The datasets presented in this study can be found in online repositories. The names of the repository/repositories and accession number(s) can be found at: https://osf.io/b5ep9/.

## Ethics Statement

The studies involving human participants were reviewed and approved by the Ethics Committee at ELTE Eötvös Loránd University (reference numbers: 2020/403 and 2021/314). This research was also conducted based on the ethical standards of Declaration of Helsinki and its later amendments. The patients/participants provided their written informed consent to participate in this study.

## Author Contributions

AA: conceptualization, methodology, formal analysis, investigation, data curation, writing (original draft), writing (review and editing), visualization, and project administration. AU: writing (review and editing) and supervision. HL: conceptualization, methodology, writing (review and editing), supervision, and funding acquisition. All authors contributed to the article and approved the submitted version.

## Funding

This research was supported by the Hungarian National Research, Development, and Innovation Office (https://nkfih.gov.hu; grant no. K131635).

## Conflict of Interest

The authors declare that the research was conducted in the absence of any commercial or financial relationships that could be construed as a potential conflict of interest.

## Publisher’s Note

All claims expressed in this article are solely those of the authors and do not necessarily represent those of their affiliated organizations, or those of the publisher, the editors and the reviewers. Any product that may be evaluated in this article, or claim that may be made by its manufacturer, is not guaranteed or endorsed by the publisher.

## Footnotes

^1^ www.mindmedia.com

^2^Akil, A. M., Oker, K., and Ujhelyi, A. (2022). Maladaptive cognitive emotion regulation strategies moderate the effects of exposure to both neutral images and depression memes on depressive mood.

^3^ www.psytoolkit.org
