# Corrigendum: Exposure to depression memes on social media increases depressive mood and it is moderated by self-regulation: Evidence from self-report and resting EEG assessments

**DOI:** 10.3389/fpsyg.2023.1146810

**Published:** 2023-02-01

**Authors:** Atakan M. Akil, Adrienn Ujhelyi, H. N. Alexander Logemann

**Affiliations:** ^1^Doctoral School of Psychology, ELTE Eötvös Loránd University, Budapest, Hungary; ^2^Institute of Psychology, ELTE Eötvös Loránd University, Budapest, Hungary

**Keywords:** depression memes, EEG, emotion regulation, frontal alpha asymmetry, internet, social media, self-regulation

In the published article, there was an error in Discussion, Paragraph 4. In the final sentence “lower depressive mood” should have been “higher depressive mood”. The corrected paragraph appears below.

The results regarding the predictive role of frontal alpha asymmetry in changes in depressive mood after exposure to depression memes can indicate several factors. Particularly, eyes closed frontal alpha asymmetry showed a similar pattern to our subjective evaluation of maladaptive emotion regulation strategies, such as difficulties in goal-directed behaviors in emotional distress and impulse control difficulties in case it is considered a lower inhibitory control, with a higher frontal alpha asymmetry score. Therefore, our results are consistent with those of studies that indicate that inhibitory control deficits result in increased processing of negative stimuli (Gotlib and Joormann, 2010; Disner et al., 2011; García-Martín et al., 2021); this is vital for emotion-related problems because it allows individuals to limit unwanted behaviors, thoughts, and emotions and provides flexibility for adapting to diverse environmental contingencies and specific goals (Anderson and Weaver, 2009). However, a higher frontal alpha asymmetry score also means less alpha activity in the left frontal cortex, that is, higher approach motivation to positive stimuli. In this case, our results were not sufficiently indicative. Specifically, eyes open frontal alpha asymmetry showed that higher avoidance/withdrawal tendency or inhibitory control, as indexed by the lower frontal alpha asymmetry scores, results in higher depressive mood after exposure to depression memes compared with neutral images, consistent with previous studies (Coan and Allen, 2004; Harmon-Jones et al., 2010).

The authors apologize for this error and state that this does not change the scientific conclusions of the article in any way. The original article has been updated.
